# Supplementary material for: Adult neurogenesis improves spatial information encoding in the mouse hippocampus
Source: Nat Commun. 2024 Jul 30;15:6410. doi: 10.1038/s41467-024-50699-x (PMC11289285; doi:10.1038/s41467-024-50699-x)
Supplement: Supplementary file 1 — Supplementary information [file 41467_2024_50699_MOESM1_ESM.pdf]

SUPPLEMENTARY INFORMATION

Adult neurogenesis improves spatial information encoding in the mouse hippocampus

M. Agustina Frechou, Sunaina S. Martin, Kelsey D. McDermott, Evan A. Huaman, Şölen Gökhan, Wolfgang A. Tomé, Ruben Coen-Cagli, J. Tiago Gonçalves

Supplementary information contents:

Supplementary Table 1

Supplementary Figure 1

Supplementary Figure 2

Supplementary Figure 3

Supplementary Figure 4

Supplementary Figure 5

Supplementary Figure 6

Supplementary Figure 7

Supplementary Figure 8

Supplementary Figure 9

Supplementary Movie 1

Supplementary Table 1

Sex of animals used in this study

Experimental Group	Females	Males
RC	2	3
EE	5	
IRR + RC	4	
IRR + EE	4	
hM4Di+	4	5
hM4Di-	2	3
retroAAV RC		3
retroAAV EE		3

Experimental Group	Mouse #	Sex
RC	M1	M
RC	M2	M
RC	M3	M
RC	M4	F
RC	M5	F
hM4Di+	M1	M
hM4Di+	M2	M
hM4Di+	M3	F
hM4Di+	M4	F
hM4Di+	M5	M
hM4Di+	M6	M
hM4Di+	M7	M
hM4Di+	M8	F
hM4Di+	M9	F
hM4Di-	M1	M
hM4Di-	M2	M
hM4Di-	M3	M
hM4Di-	M4	F
hM4Di-	M5	F

Supplementary Figure Legends

Supplementary Figure 1. Olfactory bulb neurogenesis is preserved with hippocampal focal irradiation. Typical isodose distributions for hippocampal focusing irradiation employed in this study: a) coronal plane, b) sagittal plane. c) Immunofluorescence images of the DCX-positive neurons and DAPI labelled nuclei in the olfactory bulb (left) and dentate gyrus (right) of a mouse that has undergone focal irradiation and in vivo imaging. Related to Figure 1.

Supplementary Figure 2. Immature DCX-expressing ABNs do not express jRGECO1a .
a) Representative epifluorescence image of tissue section stained with antibodies against Doublecortin (DCX, green). Immunohistochemistry was performed after in vivo imaging. jRGECO1a expression is shown in magenta and DAPI nuclear stain in blue. Note that areas with increased expression of jRGECO1a, such as the one indicated by the yellow arrowhead, are associated with a reduced numbed of DCX-expressing cells. Dotted line on top denotes location of window implant over CA1. b) Confocal optical section images of area bound by dotted square in a). Images in each column are taken at a single optical plane. DCX-expressing cells (yellow arrowheads) colocalize with gaps in jRGECO1a fluorescence. Out of 52 cell bodies imaged in 3 tissue sections from 3 different mice, not a single DCX-expressing cell co-expressed jRGECO1a. Related to Figure 1.

Supplementary Figure 3. Optimization of decoder for calcium imaging analysis. a) Photograph of two-photon imaging treadmill with different textile cues. b) Average speed of RC and EE mice on treadmill during experimental sessions. c) Comparison of percentage of decoding accuracy using different methods for filtering calcium trace data. Unfiltered raw traces, raw traces with neuropil subtraction, filtered traces using a moving average approach and window size of 100, dF/F, binary data on the location of the deconvolved spikes on the trace, traces being

represented by the amplitude of the deconvolved spikes on the trace and the deconvolved traces. Spike deconvolution was performed using suite2p based on the OASIS algorithm. d) Example of decoder accuracy using unfiltered calcium traces in EE and Irr+EE mice. e) Example of decoder accuracy using filtered calcium traces in EE and Irr+EE mice. f) Decoder accuracy on training data plotted by number of cells analyzed. g) Decoder accuracy on testing data plotted by number of cells analyzed. Related to Figure 1.

Supplementary Figure 4. Ablating adult neurogenesis reduces single-cell tuning specificity and activity. a) Distribution of single-cell tuning indices between non-irradiated and irradiated cohorts. b) Distribution of single-cell activity rates between non-irradiated and irradiated cohorts. c) Proportion of cells whose tuning curves can be well-fit (R^2>0.5) to a Von Mises function, in both non-irradiated and irradiated cohorts. Related to Figure 3.

Supplementary Figure 5. Immature hM4Di-expressing ABNs do not express jRGECO1a.
a) Representative epifluorescence image of tissue section stained with antibodies against HA-tag, which label hM4Di receptors (green). Immunohistochemistry was performed after in vivo imaging. jRGECO1a expression is shown in magenta and DAPI nuclear stain in blue. b) Confocal optical section images of. Images in each column are taken at a single optical plane. hM4Di-expressing cells (yellow arrowheads) colocalize with reduced jRGECO1a fluorescence. Related to Figure 4.

Supplementary Figure 6. Measures of population spatial information before and after acute chemogenetic silencing of ABNs
a,b) Proportion of cells whose tuning curves can be well-fit (R^2>0.5) to a Von Mises function, before and after injection of CNO. c) Accuracy in decoding position of mouse on treadmill from calcium traces (Baseline vs CNO: p=0.31, n = 4 mice, paired t-test). d) Population Fisher

information (d'^2) before and after silencing of a cohort of ABNs with CNO (Baseline vs CNO: $p=0.0071$, $n = 4$ mice, paired t-test). Related to Figure 4.

Supplementary Figure 7. EE increases DG single-cell activity and spatial information in mice injected with a retro AAV virus in CA3.

a) Experimental timeline b) Activity (RC vs EE: $p = 0.00143$, $n_{RC} = 3$ mice, 49 neurons, $n_{EE} = 4$ mice, 69 neurons) and c) Fisher Information (RC vs EE: $p < 1/10000$, $n_{RC} = 3$ mice, 49 neurons, $n_{EE} = 4$ mice, 69 neurons) in mice injected with a retro AAV virus in CA3. d) Tuning Index (RC vs EE: $p = 0.36$, $n_{RC} = 3$ mice, 49 neurons, $n_{EE} = 4$ mice, 69 neurons)

Supplementary Figure 8. CA3 injection of retrograde AAV vectors exclusively targets mature ABNs.

a) Experimental design: the DG of wild-type mice was injected with a RV-GFP vector that exclusively labels cells undergoing cell division at the time of injection. All animals received a second injection of retrograde AAV encoding jRGECO1a in hippocampal area CA3, either 17 (Group 1) or 25 days (Group 2) after the first injection. Brain tissue was collected 3.5 weeks later, in agreement with timeline used for in vivo imaging. GFP-labeled cells in the DG were either 6 weeks (Group 1) or 7 weeks (Group 2) post-mitosis at the time of perfusion. b) Representative confocal optical sections displaying RV-GFP labeled ABNs (green), jRGECO1a expression (magenta) and DAPI nuclear stain (blue). Group 1 GFP-labeled ABNs (yellow arrowheads) did not co-express jRGECO1a, indicating that they were not infected with retrograde AAV, even though neighboring cells displayed strong jRGECO1a fluorescence. Conversely, Group 2 GFP-labeled ABNs consistently co-expressed jRGECO1a, indicating that when the injection occurs at 25 days post-mitosis these ABNs do undergo retrograde AAV infection and express the calcium indicator.

Supplementary Figure 9. Microglia in irradiated tissue show morphological hallmarks of increased inflammation.

a) Representative fluorescence images of EE and Irr+EE tissue sections of stained with antibodies against Iba1, which label microglia (green). jRGECO1a expression is shown in red. Images are maximum projection of 10 µm confocal stack. b) Example of reconstructed microglia from EE and Irr+EE mice. Microglia in irradiated tissue have fewer branches that are shorter than their non-irradiated counterparts. c) Sholl analysis of microglia process complexity. Number of intersections of concentric Sholl spheres at varying distances from the cell body (EE vs Irr+EE: n_{EE} = 2 mice, 18 cells, n_{Irr+EE} = 2 mice, 18 cells, shaded area is SEM). D) Location of peak of Sholl intersections measured as a distance from cell body (* p=0.013, Mann-Whitney U-test) E) Total number of Sholl intersections (****p < 1/10000, Mann-Whitney U-test). F) Total branch length (****p < 1/10000, Mann-Whitney U-test).

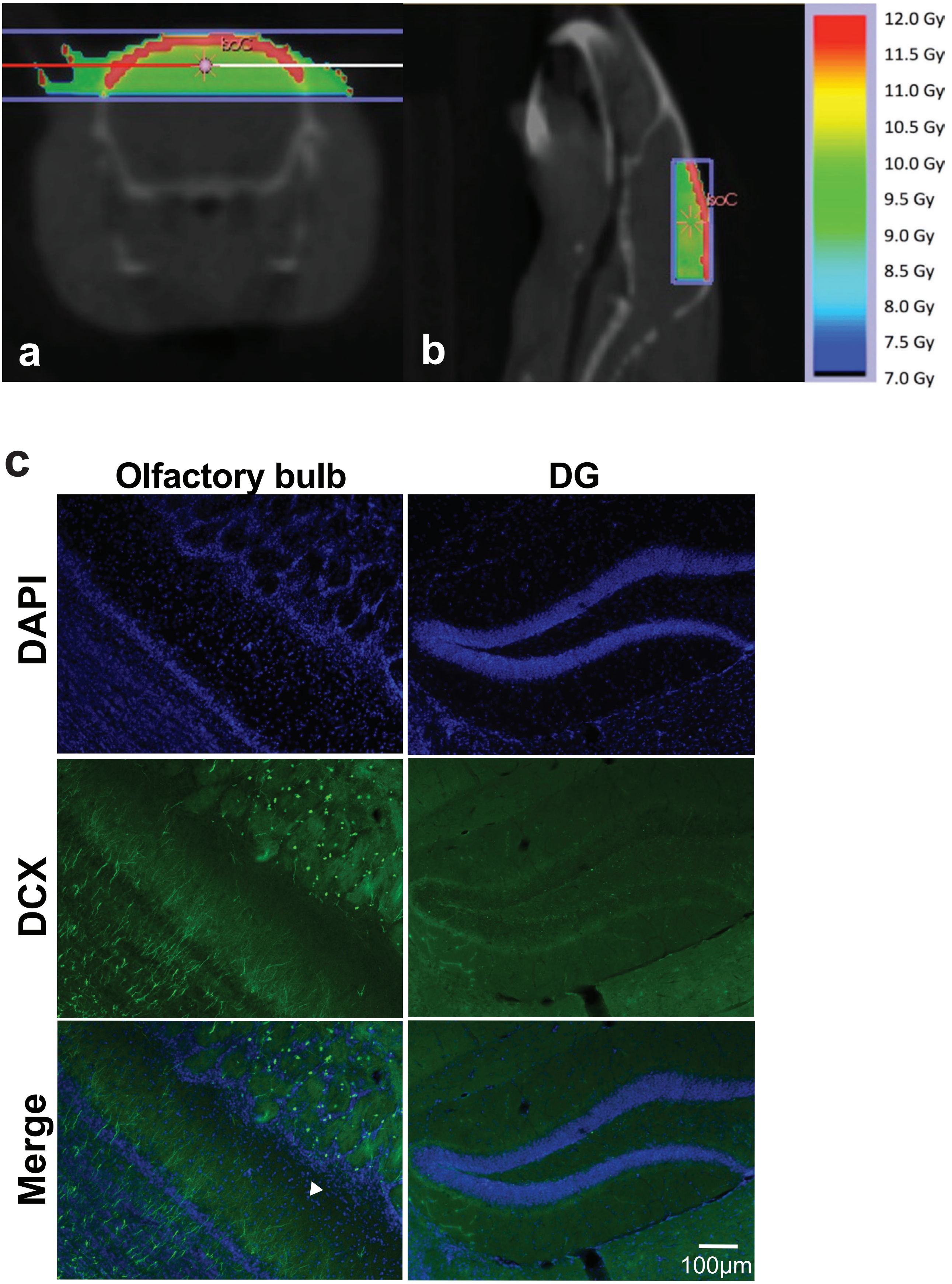

Supplementary Figure 1

Supplementary Figure 2

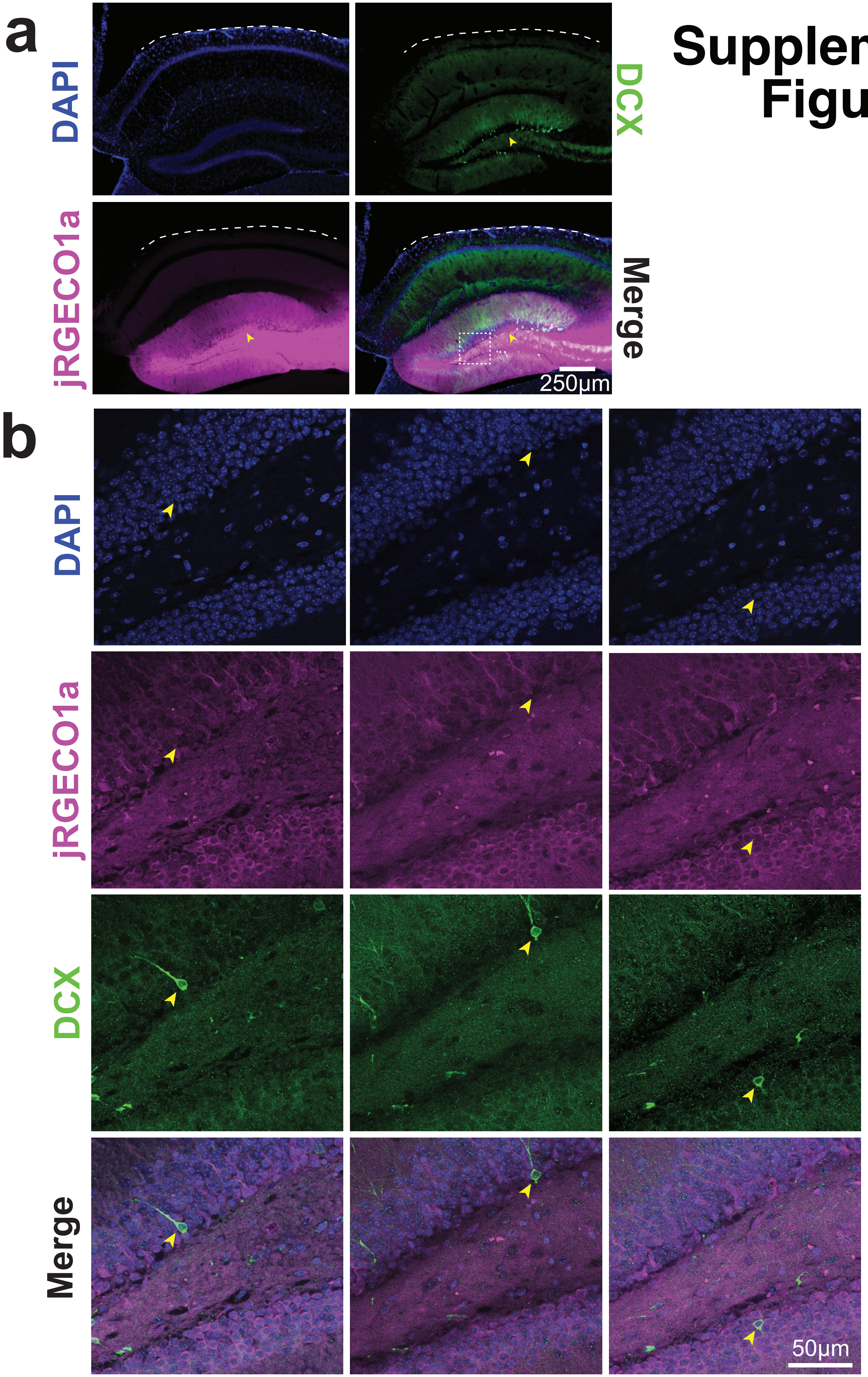

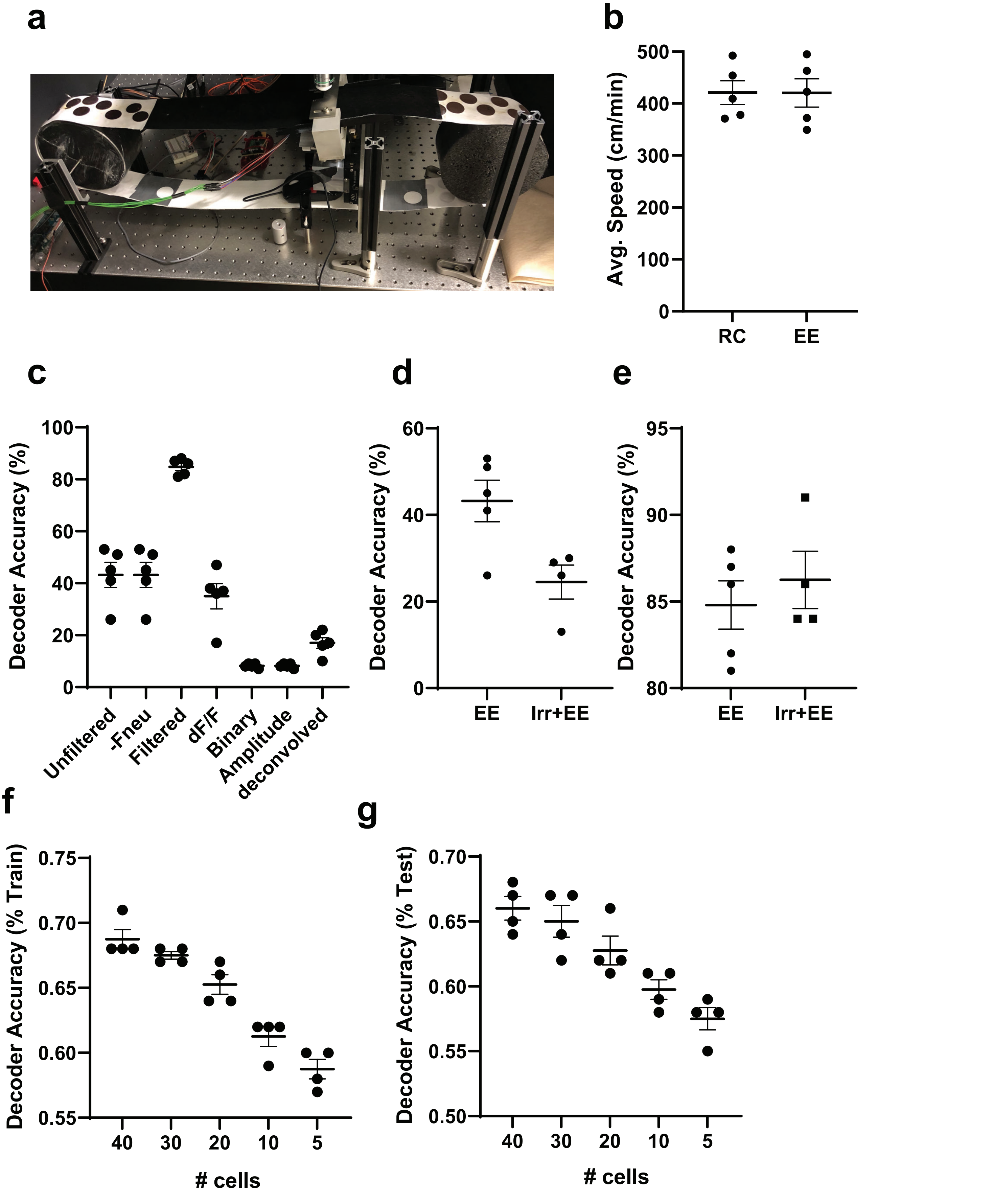

Supplementary Figure 3

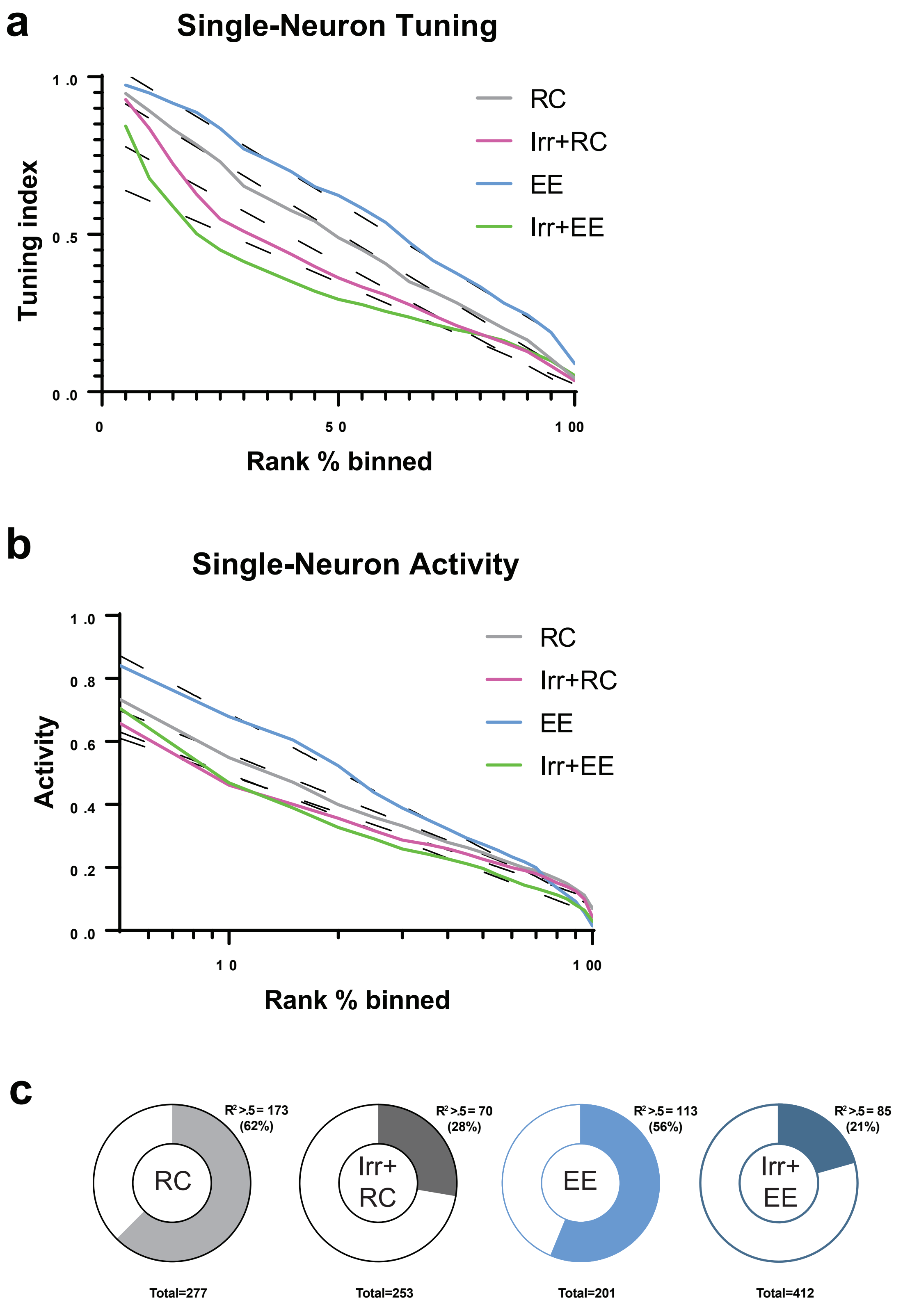

Supplementary Figure 4

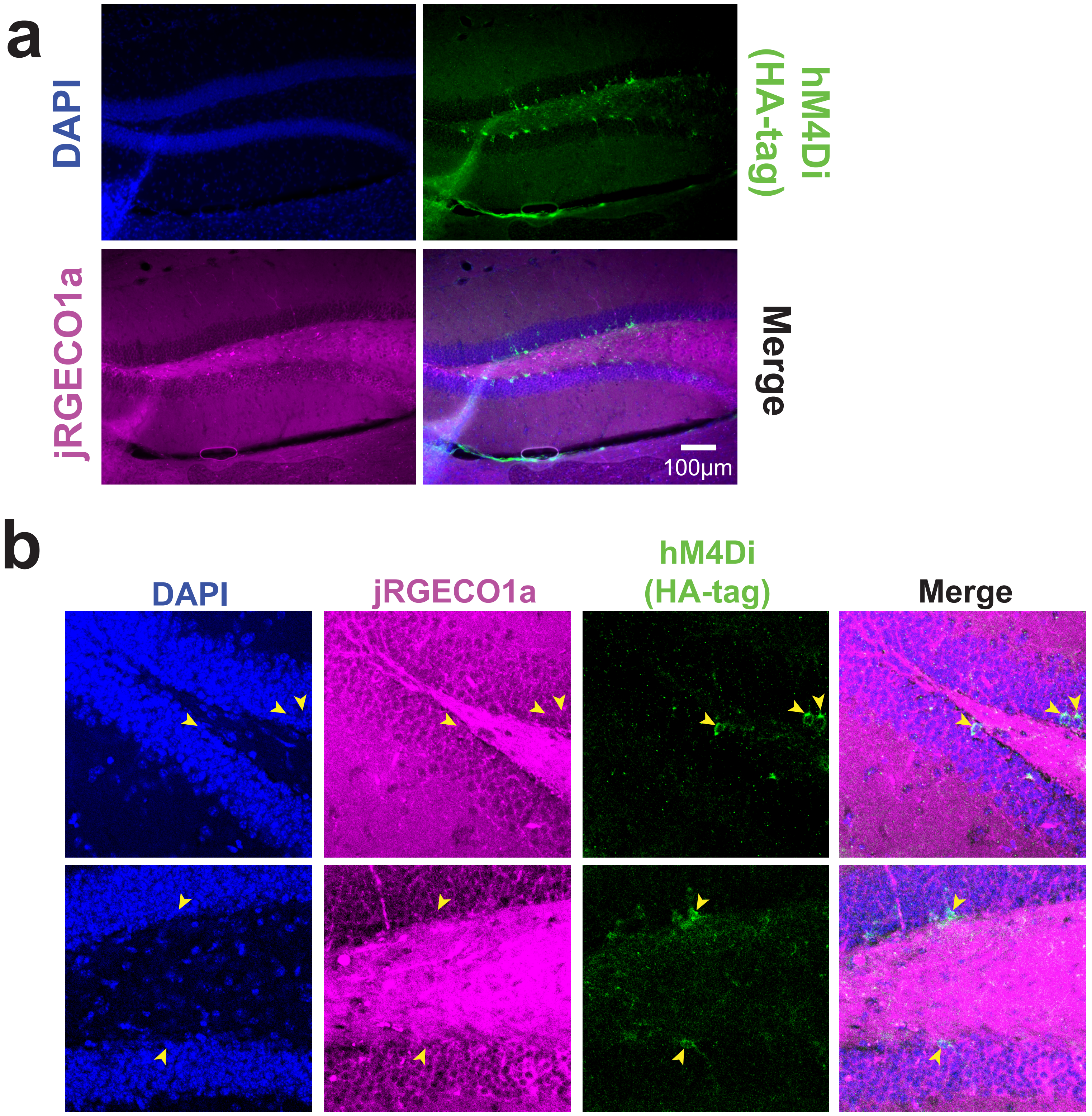

Supplementary Figure 5

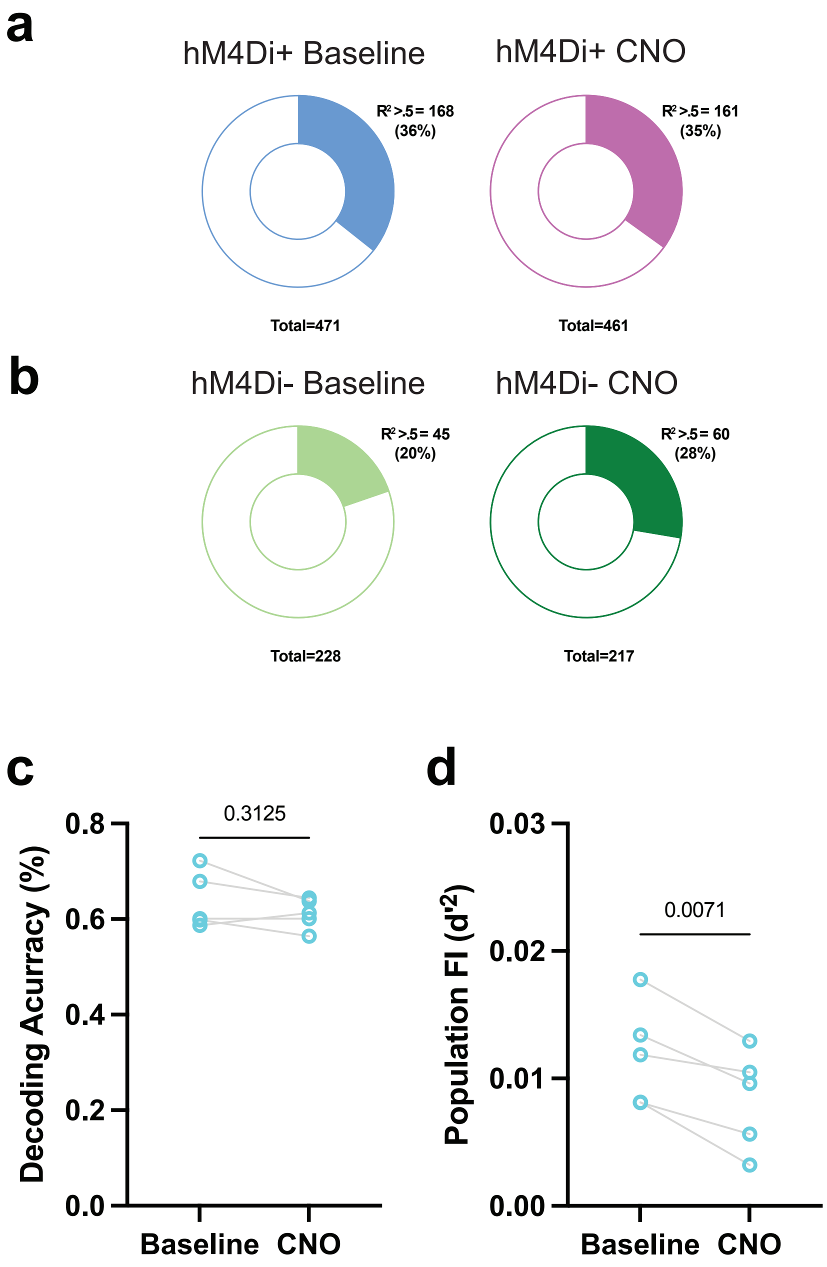

Supplementary Figure 6

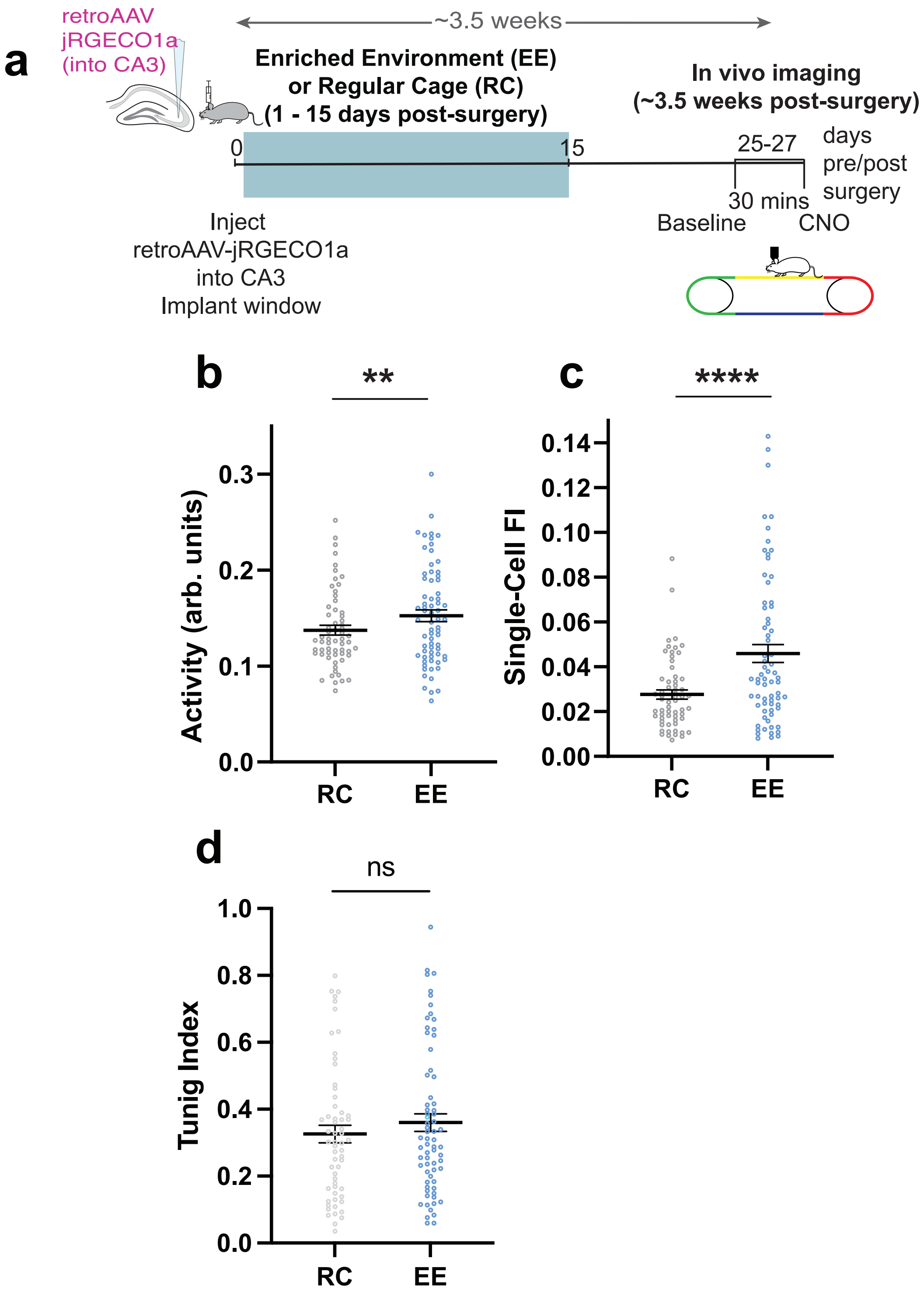

Supplementary Figure 7

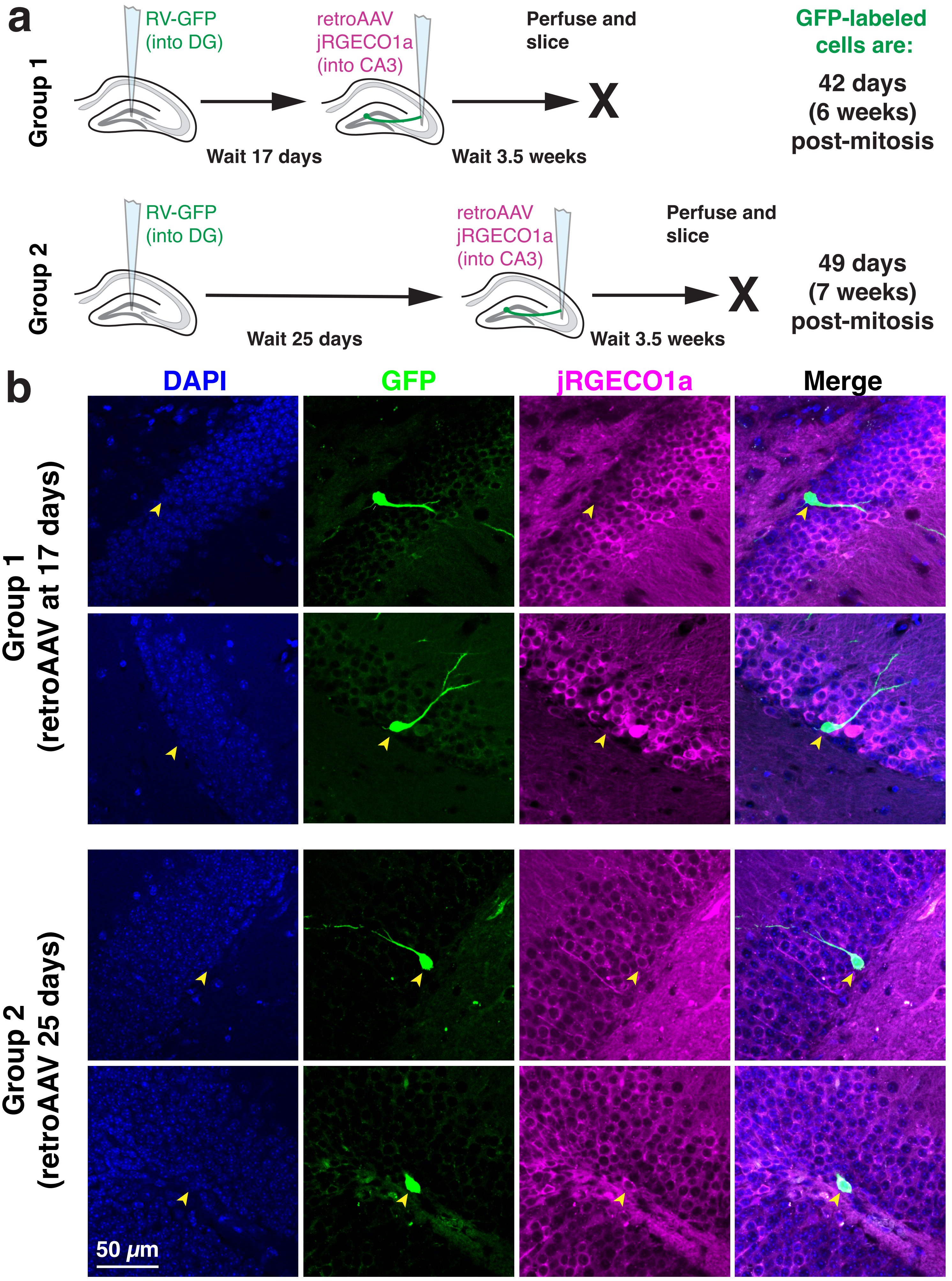

Supplementary Figure 8

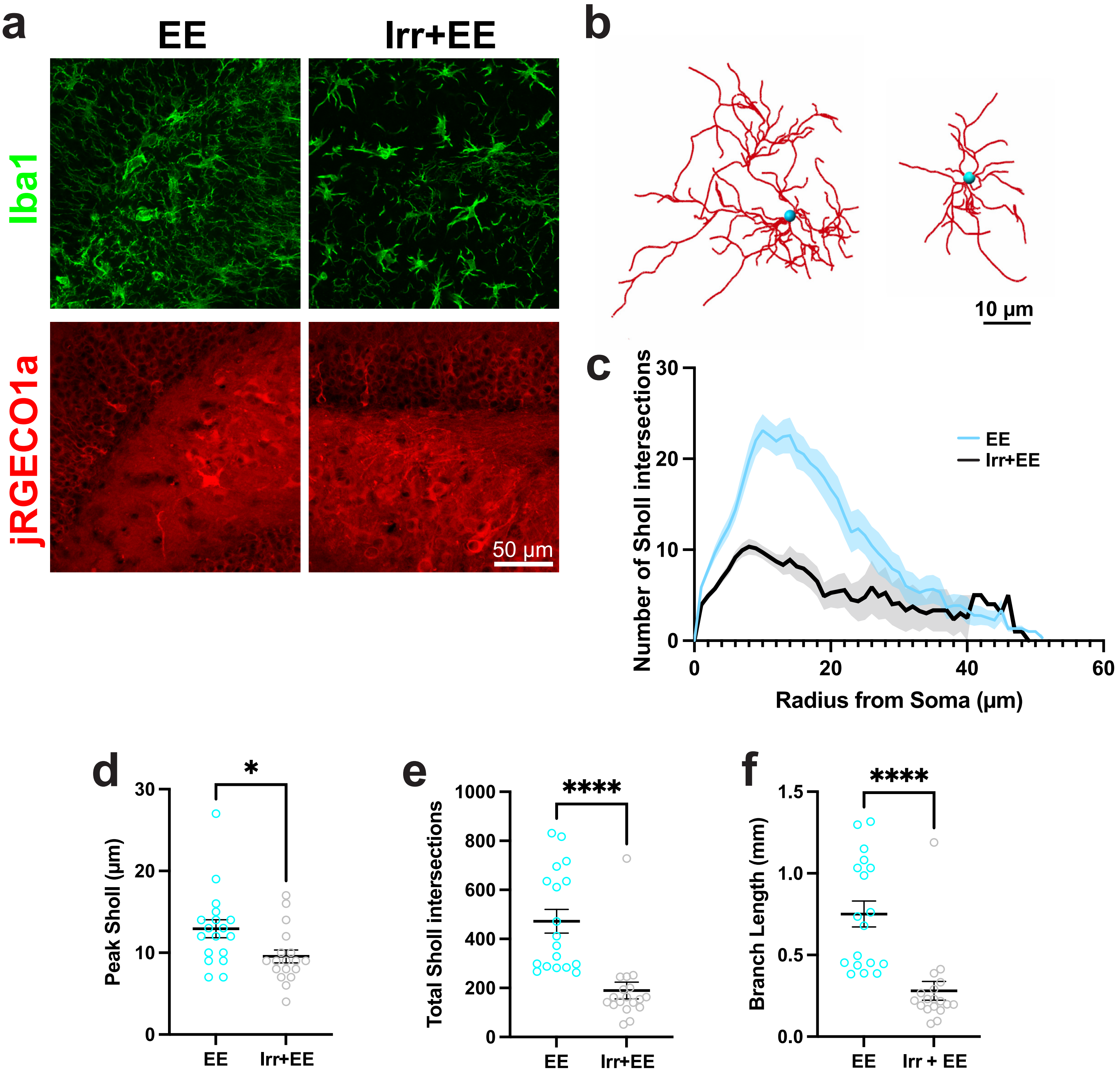

Supplementary Figure 9
