# Supplementary material for: Adult neurogenesis improves spatial information encoding in the mouse hippocampus
Source: Nat Commun. 2024 Jul 30;15:6410. doi: 10.1038/s41467-024-50699-x (PMC11289285; doi:10.1038/s41467-024-50699-x)
Supplement: Supplementary file 3 — Description of Additional Supplementary Files [file 41467_2024_50699_MOESM3_ESM.pdf]

DESCRIPTION OF ADDITIONAL SUPPLEMENTARY FILES

Adult neurogenesis improves spatial information encoding in the mouse hippocampus

M. Agustina Frechou, Sunaina S. Martin, Kelsey D. McDermott, Evan A. Huaman, Şölen Gökhan, Wolfgang A. Tomé, Ruben Coen-Cagli, J. Tiago Gonçalves

Supplementary Movie 1. Example DG calcium imaging movie

Example excerpt of calcium imaging movie recorded from the DG of a mouse that is walking head-fixed on a treadmill. Movie was registered and every 10 frames were averaged for display purposes. Field of view is 343.6 x 343.6 µm, total duration is 250 s.
