# Supplementary material for: Adult neurogenesis improves spatial information encoding in the mouse hippocampus
Source: Nat Commun. 2024 Jul 30;15:6410. doi: 10.1038/s41467-024-50699-x (PMC11289285; doi:10.1038/s41467-024-50699-x)
Supplement: Supplementary file 5 — Reporting Summary [file 41467_2024_50699_MOESM5_ESM.pdf]

nature portfolio

Corresponding author(s): J. Tiago Gonçalves

Last updated by author(s): Jun 7, 2024

Reporting Summary

Nature Portfolio wishes to improve the reproducibility of the work that we publish. This form provides structure for consistency and transparency in reporting. For further information on Nature Portfolio policies, see our Editorial Policies and the Editorial Policy Checklist.

Statistics

For all statistical analyses, confirm that the following items are present in the figure legend, table legend, main text, or Methods section.

n/a	Confirmed	
☐	☒	The exact sample size (*n*) for each experimental group/condition, given as a discrete number and unit of measurement
☐	☒	A statement on whether measurements were taken from distinct samples or whether the same sample was measured repeatedly
☐	☒	The statistical test(s) used AND whether they are one- or two-sided<br>*Only common tests should be described solely by name; describe more complex techniques in the Methods section.*
☐	☒	A description of all covariates tested
☐	☒	A description of any assumptions or corrections, such as tests of normality and adjustment for multiple comparisons
☐	☒	A full description of the statistical parameters including central tendency (e.g. means) or other basic estimates (e.g. regression coefficient) AND variation (e.g. standard deviation) or associated estimates of uncertainty (e.g. confidence intervals)
☐	☒	For null hypothesis testing, the test statistic (e.g. *F*, *t*, *r*) with confidence intervals, effect sizes, degrees of freedom and *P* value noted<br>*Give P values as exact values whenever suitable.*
☒	☐	For Bayesian analysis, information on the choice of priors and Markov chain Monte Carlo settings
☐	☒	For hierarchical and complex designs, identification of the appropriate level for tests and full reporting of outcomes
☒	☐	Estimates of effect sizes (e.g. Cohen's *d*, Pearson's *r*), indicating how they were calculated

Our web collection on statistics for biologists contains articles on many of the points above.

Software and code

Policy information about availability of computer code

Data collection	Data was collected with the proprietary software from microscope manufacturer. We used a Thorlabs Bergamo microscope. The acquisition software was ThorImage 4.0 and ThorSync 4.0, as described in the methods section.
Data analysis	Image segmentation and selection of ROIs to extract calcium traces of individual cells was done with Suite2p version 0.9.2. The remaining data analysis was done with custom software and a link to the source code was included.

For manuscripts utilizing custom algorithms or software that are central to the research but not yet described in published literature, software must be made available to editors and reviewers. We strongly encourage code deposition in a community repository (e.g. GitHub). See the Nature Portfolio guidelines for submitting code & software for further information.

Data

Policy information about availability of data

All manuscripts must include a data availability statement. This statement should provide the following information, where applicable:

- Accession codes, unique identifiers, or web links for publicly available datasets
- A description of any restrictions on data availability
- For clinical datasets or third party data, please ensure that the statement adheres to our policy

All the data generated in this study have been deposited in the Zenodo database and links to individual datasets can be accessed at https://github.com/GoncalvesLab/Frechou-et-al.-datasets (doi:10.5281/zenodo.10985968).

The analysis code used in this study has been deposited on Github and can be accessed at https://github.com/GoncalvesLab/Frechou-et-al-Neurogenesis (doi: 10.5281/zenodo.10986236).

Research involving human participants, their data, or biological material

Policy information about studies with human participants or human data. See also policy information about sex, gender (identity/presentation), and sexual orientation and race, ethnicity and racism.

Reporting on sex and gender	No human data or human biological materials were used in this study.
Reporting on race, ethnicity, or other socially relevant groupings	*Please specify the socially constructed or socially relevant categorization variable(s) used in your manuscript and explain why they were used. Please note that such variables should not be used as proxies for other socially constructed/relevant variables (for example, race or ethnicity should not be used as a proxy for socioeconomic status).*<br>*Provide clear definitions of the relevant terms used, how they were provided (by the participants/respondents, the researchers, or third parties), and the method(s) used to classify people into the different categories (e.g. self-report, census or administrative data, social media data, etc.)*<br>*Please provide details about how you controlled for confounding variables in your analyses.*
Population characteristics	*Describe the covariate-relevant population characteristics of the human research participants (e.g. age, genotypic information, past and current diagnosis and treatment categories). If you filled out the behavioural & social sciences study design questions and have nothing to add here, write "See above."*
Recruitment	*Describe how participants were recruited. Outline any potential self-selection bias or other biases that may be present and how these are likely to impact results.*
Ethics oversight	*Identify the organization(s) that approved the study protocol.*

Note that full information on the approval of the study protocol must also be provided in the manuscript.

Field-specific reporting

Please select the one below that is the best fit for your research. If you are not sure, read the appropriate sections before making your selection.

☒ Life sciences ☐ Behavioural & social sciences ☐ Ecological, evolutionary & environmental sciences

For a reference copy of the document with all sections, see nature.com/documents/nr-reporting-summary-flat.pdf

Life sciences study design

All studies must disclose on these points even when the disclosure is negative.

Sample size	Estimating statistical power for some of the analyses we used (eg. linear decoders, nested designs) can be complex. For all main figures we used a minimum of 4 mice per experimental group as is customary in the field. This was sufficient to find differences in decoding accuracy, Fisher Information and tuning.
Data exclusions	Successfully acquired data was not excluded but some animals did not result in useful imaging data due to low expression, opaque implants etc. Linear decoding was used with a minimum threshold of 42 cells and animals that did not have 42 active cells during a recording session were excluded from these analyses.
Replication	Findings of increased tuning/Fisher information in mice exposed to EE were first noted in pilot experiments and were consistently replicated.<br>The number of biological replicates for each experimental group is indicated in the figure legends.<br>All representative figures were depict typical results of experiments with several biological replicates (described in Statistics and Reproducibility section: Fig. 1c: nRC = 5 mice, nIrr+RC = 4 mice. Fig. 4b: n = 5 mice)
Randomization	Litter mates were assigned to different experimental groups in a random manner. For hM4Di silencing experiments, control animals were litter mates that lacked the Cre allele.
Blinding	In hM4Di silencing experiments the investigator was fully blinded to experimental group/ genotype. In other experiments, the same investigator performed different manipulations and could therefore be aware of experimental group.

Reporting for specific materials, systems and methods

We require information from authors about some types of materials, experimental systems and methods used in many studies. Here, indicate whether each material, system or method listed is relevant to your study. If you are not sure if a list item applies to your research, read the appropriate section before selecting a response.

Materials & experimental systems

n/a	Involved in the study
☐	☒ Antibodies
☒	☐ Eukaryotic cell lines
☒	☐ Palaeontology and archaeology
☐	☒ Animals and other organisms
☒	☐ Clinical data
☒	☐ Dual use research of concern
☐	☐ Plants

Methods

n/a	Involved in the study
☒	☐ ChIP-seq
☒	☐ Flow cytometry
☒	☐ MRI-based neuroimaging

Antibodies

Antibodies used	All antibodies are described in methods section under "Immunohistochemistry and imaging of fixed tissue". Doublecortin (DCX): CST 14802S, 1:800 or Abcam ab18723, 1:500. HA-Tag: CST 3724S, 1:800. Iba1: Fujifilm Wako 019-19741, 1:500. Secondary antibodies Alexa 488 (A11034), Alexa 568 (A11011), or Alexa 633 (A21070) goat anti-rabbit (Invitrogen) 1:500
Validation	All antibodies have been extensively validated by the manufacturers for immunohistochemistry in frozen, PFA-fixed sections at the concentrations that we used. They were also used in several publications, as detailed in the product pages below:<br><br>Doublecortin (DCX) - CST 14802S: https://www.cellsignal.com/products/primary-antibodies/doublecortin-a8l1u-rabbit-mab/14802<br><br>Doublecortin (DCX) - Abcam ab18723: https://www.abcam.com/products/primary-antibodies/doublecortin-antibody-ab18723.html<br><br>HA-Tag - CST 3724S: https://www.cellsignal.com/products/primary-antibodies/ha-tag-c29f4-rabbit-mab/3724 (This antibody is one of the recommended by Dr. Bryan Roth, who developed hM4Di)<br><br>Iba1 - Fujifilm Wako 019-19741: https://labchem-wako.fujifilm.com/us/product/detail/W01W0101-1974.html

Animals and other research organisms

Policy information about studies involving animals; ARRIVE guidelines recommended for reporting animal research, and Sex and Gender in Research

Laboratory animals	Mouse strains and housing information are reported in Methods under the "Animals" section. All mice were acquired from Jackson Labs or bred in-house from progenitors that were acquired from Jackson Labs. The stock numbers for each mouse line are provided in the text: C57BL6/J (Jackson Labs Stock #664) and, for chemogenetic silencing experiments, the offspring of hM4Di-Dreadd (Jackson Labs #26219) and Ascl1-Cre-ERT2 mice (Jackson Labs #12882). All mice were kept on a 12h light/dark cycle under standard environmental conditions (18-23o C, 40-60% relative humidity) and were allowed standard chow and water ad libitum.
Wild animals	Not used.
Reporting on sex	The sex of animals is stated in Supplementary Table 1 (which is referred to in Methods section). Most experimental groups used female animals due to the constraints of housing non-litter mate males in enriched environments. However, chemogenetic silencing experiments used both male and female mice. We did not test for differences between male and female mice due to having an insufficient sample size of each sex.
Field-collected samples	Not used.
Ethics oversight	Reported in Methods under "Animals" section. All procedures were done in accordance with a protocol approved by the Institutional Animal Care and Use Committee of the Albert Einstein College of Medicine (Protocol #: 00001197).

Note that full information on the approval of the study protocol must also be provided in the manuscript.

Plants

Seed stocks	*Report on the source of all seed stocks or other plant material used. If applicable, state the seed stock centre and catalogue number. If plant specimens were collected from the field, describe the collection location, date and sampling procedures.*
Novel plant genotypes	*Describe the methods by which all novel plant genotypes were produced. This includes those generated by transgenic approaches, gene editing, chemical/radiation-based mutagenesis and hybridization. For transgenic lines, describe the transformation method, the number of independent lines analyzed and the generation upon which experiments were performed. For gene-edited lines, describe the editor used, the endogenous sequence targeted for editing, the targeting guide RNA sequence (if applicable) and how the editor was applied.*
Authentication	*Describe any authentication procedures for each seed stock used or novel genotype generated. Describe any experiments used to assess the effect of a mutation and, where applicable, how potential secondary effects (e.g. second site T-DNA insertions, mosiacism, off-target gene editing) were examined.*
